# Atomic-level interface engineering enables efficient and durable acidic hydrogen evolution of osmium at large current densities

**DOI:** 10.1039/d5sc09741j

**Published:** 2026-02-10

**Authors:** Qianyi Lin, Jun Yu, Mansheng Liao, Weidong Liang, Yayun Hong, Huiqi Li, Zhongxin Song, Lei Zhang

**Affiliations:** a College of Chemistry and Environmental Engineering, Shenzhen University Shenzhen 518071 China yujun@szu.edu.cn lei.zhang@szu.edu.cn

## Abstract

Osmium (Os), the least expensive member of the platinum-group metals, has emerged as a promising alternative to Pt-based catalysts for the hydrogen evolution reaction (HER). However, Os-based electrocatalysts still suffer from poor stability under acidic conditions, despite recent efforts to mitigate H* over-adsorption for improved intrinsic activity. Here, we design a porous CeO_2_ support that enables the atomic dispersion of Os, forming an Os single-atom catalyst (Os_SA_–CeO_2_). Unlike traditional flat-film supports, the porous CeO_2_ architecture prevents Os aggregation and achieves 100% interfacial anchoring of Os atoms. The resulting strong electronic coupling enables tight anchoring of Os and activates the CeO_2_ matrix with abundant oxygen vacancies, which facilitate H_2_O dissociation to sustainably supply protons for rapid consumption at large current densities. Also, the generated OH* species are adsorbed by the oxygen vacancies, thus preventing the Os sites from oxidative dissolution. As a result, Os_SA_–CeO_2_ exhibits over 500 h of durability at 100 mA cm^−2^ without performance decay—surpassing all previously reported Os-based HER catalysts. This work provides a general strategy for achieving complete interfacial anchoring of active metal atoms to enhance catalytic stability without sacrificing activity through support activation.

## Abstract 2

Porous CeO_2_ achieves atomic dispersion of Os and strong interfacial coupling, stabilizing Os atoms and activating CeO_2_ with oxygen vacancies for ultra-stable acidic hydrogen evolution.

## Graphical abstract

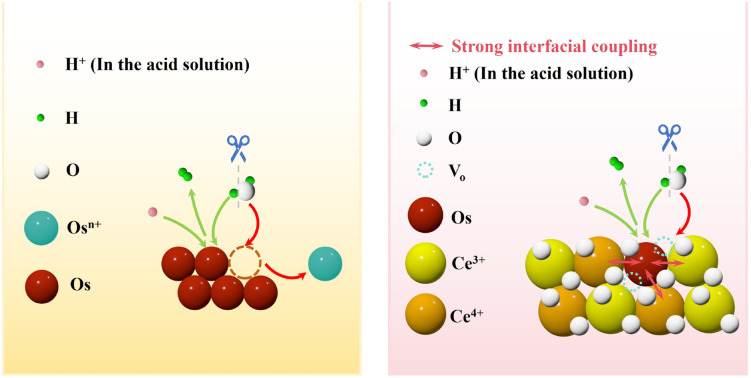

## Introduction

The transition to sustainable and non-polluting energy sources has become increasingly urgent amid the global energy, environmental, and geopolitical crises. Among various clean energy technologies, “green hydrogen” has attracted particular attention as a zero-carbon fuel to play a dominant role in the future hydrogen economy.^1–4^ Electrocatalytic water splitting powered by renewable energy sources (*e.g.*, solar and wind) represents one of the most promising routes for producing high-purity green hydrogen.^5–7^ As the cost of renewable electricity continues to decline, the price, activity, and durability of electrocatalysts for the hydrogen evolution reaction (HER) are becoming the primary factors determining the overall economic feasibility of this process.^6,8–10^

Platinum (Pt)-based catalysts exhibit the highest HER activity due to their optimal hydrogen adsorption free energy. However, the high cost and scarcity of Pt significantly hinder their large-scale application.^6,11–13^ Osmium (Os), the least expensive member of the platinum group metals (PGMs), has recently emerged as a potential alternative to Pt-based catalysts.^14–17^ A fundamental challenge for Os-based HER catalysts is their tendency to over-adsorb hydrogen intermediates (H*), which results in inherently low HER activity.^18,19^ To address this, several strategies have been developed to tune the electronic structure of Os and modulate its hydrogen adsorption behavior, such as supporting Os nanoparticles on TiO_2_,^20^ constructing heterostructures like Os–OsSe_2_,^15,18,19^ and introducing anion doping.^21^ While these approaches have improved catalytic performance, achieving long-term stability, particularly at high current densities (≥100 mA cm^−2^), remains a major challenge.^15,18,19^

The instability of Os-based catalysts primarily originates from nanoparticle aggregation *via* Ostwald ripening and the easily fluctuating valence states of Os under the harsh conditions of the HER.^14,22^ At large current densities, oxidative dissolution of Os becomes more pronounced due to the rapid consumption of local H^+^ and the accumulation of newly generated OH^−^ species derived from H_2_O dissociation.^23^ Anchoring Os onto reducible metal oxides, such as CeO_2_, offers a promising pathway to enhance stability. The facile Ce^4+^/Ce^3+^ redox cycling and high-order f orbitals of CeO_2_ facilitate strong electronic coupling with supported metal atoms.^24–27^ Moreover, the oxyphilic nature of Ce and the ease of oxygen vacancy formation endow CeO_2_ with a strong affinity for OH^−^ species,^23,28–30^ which can effectively suppress the adsorption of OH^−^ on Os and thus prevent its oxidative dissolution. To achieve these benefits, each Os atom must directly interact with neighboring Ce atoms *i.g.* achieving atomic dispersion of Os on CeO_2_.^31–33^ However, the fabrication of this precise architectural structure presents a great challenge and it requires the investigation of the thermodynamic stability properties of isolated Os atoms.

In this work, we first investigated the structural stability and hydrogen adsorption behavior of atomically dispersed Os on CeO_2_ (Os_SA_–CeO_2_) using density functional theory (DFT) calculations. The results reveal that Os atoms can be strongly anchored within the CeO_2_ lattice, while their excessive H* adsorption is effectively mitigated. Guided by these insights, we synthesized Os_SA_–CeO_2_*via* a two-step electrodeposition method. A CeO_2_ thin film was first deposited onto a conductive carbon fiber substrate, followed by O_2_ annealing to create a porous structure that promotes atomic Os dispersion. This architecture introduces abundant oxygen vacancies and induces lattice compression, which shortens the distance between Os atoms and adjacent vacancies. These features facilitate H_2_O dissociation and the subsequent migration of H* species to neighboring Os sites, providing a continuous hydrogen source for proton consumption at large current densities. Meanwhile, the generated OH* intermediates are preferentially adsorbed by the oxygen vacancies rather than the Os sites, effectively suppressing oxidative dissolution. Consequently, Os_SA_–CeO_2_ exhibits a remarkably low overpotential of 97 mV and over 500 h of durability at 100 mA cm^−2^ with negligible degradation, surpassing nearly all previously reported Os-based HER catalysts.

## Results and discussion

### DFT calculations for material design

**Fig. 1:**
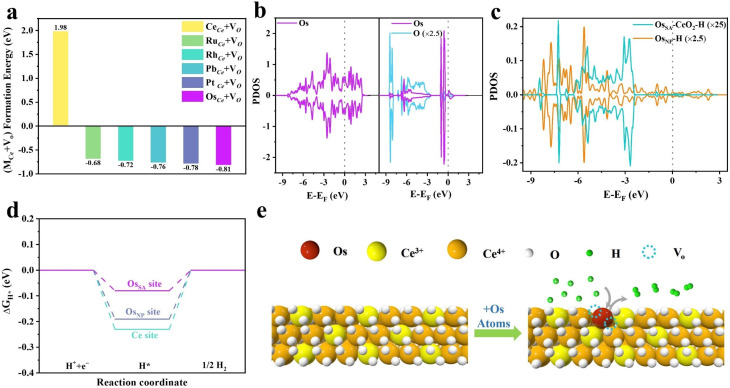
Theoretical calculations illustrating the strong interfacial coupling of Os SAs in CeO_2_. (a) Calculated formation energies of M_Ce_ + V_O_ (M stands for the substituted atom for Ce). PDOS for (b) Os 5d orbital of Os_NP_ (left) and Os_SA_–CeO_2_ (right) and (c) H 1s orbital of adsorbed H. (d) Gibbs free energy diagram for the HER in acid media on Os_NP_ (blue line) and Os_SA_–CeO_2_ (purple and cyan lines). (e) Schematic mechanism of proton adsorption on the Os site leading to H_2_ generation.

To assess the feasibility of Os incorporation into CeO_2_, we calculated the formation energies (FEs) of Os substituting Ce (Os_Ce_) and Os_Ce_ accompanied by an oxygen vacancy (Os_Ce_ + V_O_). The FE of Os_Ce_ on the CeO_2_ (111) surface is 0.64 eV (Fig. S1), whereas that of Os_Ce_ + V_O_ is −0.81 eV (Fig. 1a). The significantly lower FEs of Os_Ce_ + V_O_ compared to Os_Ce_ indicate that oxygen vacancies form readily in Os-doped CeO_2_. Compared to other investigated platinum-group metals, Os_Ce_ + V_O_ exhibits the lowest FE, demonstrating the high structural stability of atomically dispersed Os in CeO_2_ (Os_SA_–CeO_2_).

The strong anchoring of Os atoms originates from pronounced interfacial electronic coupling with the CeO_2_ lattice, as revealed by DFT calculations. For metallic Os nanoparticles (Os_NP_), the projected density of states (PDOS) displays delocalized Os 5d orbitals with occupied states at the Fermi level (Fig. 1b, left), showing metallic behavior. Upon Os doping into CeO_2_, the 5d orbitals become localized, and unoccupied states above the Fermi level nearly vanish (Fig. 1b, right). This reflects charge redistribution and strong hybridization between Os and CeO_2_. The modified electronic structure of Os in Os_SA_–CeO_2_ brings in different hydrogen adsorption behavior (Fig. 1c). The energy range of its hybridization with the H 1s orbital is approximately −6.3 to −2.3 eV for Os_SA_–CeO_2_ compared to −8 to −4 eV for Os_NP_, indicating the weaker interaction between Os and H in Os_SA_–CeO_2_. The Gibbs free energy diagram for the HER (Fig. 1d) further supports this result. The calculated Δ*G*_H*_ values for the Ce site in Os_SA_–CeO_2_ and Os site in Os_NP_ are −0.23 eV and −0.19 eV, respectively, implying facile H adsorption. However, this hinders the desorption of H*, thereby limiting the formation of H_2_. In contrast, the Os site in Os_SA_–CeO_2_ exhibits a moderate Δ*G*_H*_ of −0.08 eV and a shortened H–H coupling distance (1.60 Å *vs.* 1.97 Å for Os_NP_, Fig. S2), suggesting optimized hydrogen binding and improved HER kinetics. Overall, atomic Os incorporation into CeO_2_ yields a stable configuration with balanced H* adsorption energy and robust structural anchoring (Fig. 1e).

### Material synthesis and structural characterization

**Fig. 2:**
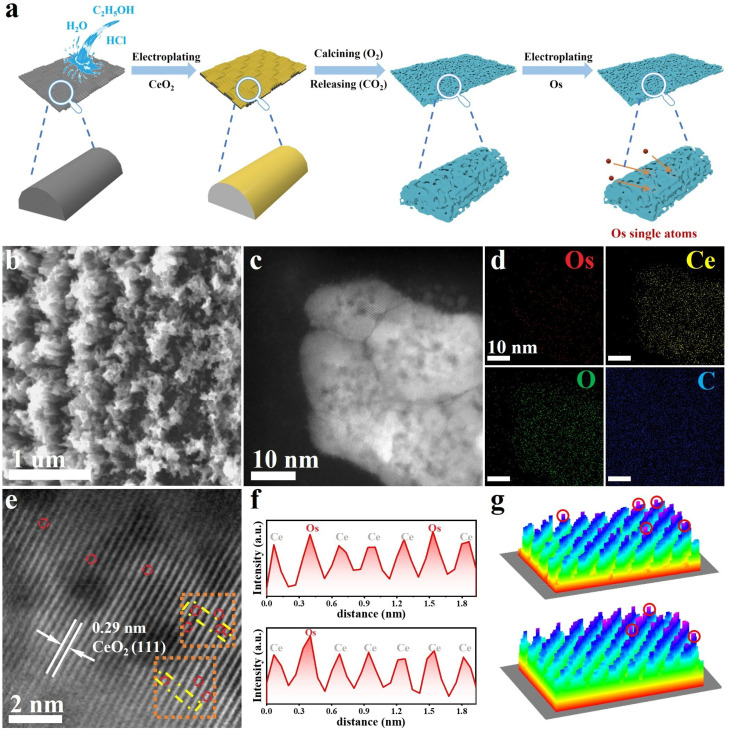
Synthesis and morphological characterization of Os SAs anchored on porous CeO_2_ (Os_SA_–CeO_2_). (a) Schematic illustration of the synthesis route for Os SAs dispersed on porous CeO_2_. (b) SEM image of Os_SA_–CeO_2_. (c) HAADF-STEM image and (d) corresponding elemental mapping images of Os_SA_–CeO_2_. (e) High-resolution HAADF-STEM image of Os_SA_–CeO_2_. (f) Two-dimensional atomic distribution map corresponding to the yellow region highlighted in (e). (g) Three-dimensional atomic distribution map corresponding to the orange region highlighted in (e).

Guided by the theoretical predictions, we synthesized the Os_SA_–CeO_2_ sample consisting of Os SAs dispersed in the CeO_2_ matrix (Fig. 2a). Compared with the dense film structure of CeO_2_ (Fig. S3), the O_2_ post-treatment induced CO_2_ release from the carbon substrate, generating a porous structure of CeO_2_–O_2_ (Fig. S4) that was preserved in Os_SA_–CeO_2_ (Fig. 2b). The aberration-corrected high-angle annular dark field scanning transmission electron microscopy (HAADF-STEM) image (Fig. 2c) reveals abundant dark regions within the sheet-like aggregates, corresponding to the voids seen in the scanning electron microscopy (SEM) image (Fig. 2b). Elemental mapping (Fig. 2d and Table S1) and high-resolution STEM imaging (Fig. 2e) confirm the sheet-like CeO_2_ structure with atomically dispersed Os. The reduced lattice spacing (0.29 nm *vs.* 0.31 nm for pristine CeO_2_) indicates lattice compression due to Os incorporation. Fig. 2f shows the 2D atom distribution map of the yellow area framed in Fig. 2e. The atoms with higher intensity are Os single atoms, and those with weaker intensity are Ce atoms. The 3D atom distribution map of the orange area in Fig. 2e also demonstrates the successful preparation of Os single atoms.

Reference samples (Os_NP_/CeO_2_ and Os_NP_) were prepared *via* the same electrodeposition route for Os (Fig. S5–S6). Compared to Os_SA_–CeO_2_, the electrodeposited CeO_2_ film was annealed in Ar instead of O_2_ in Os_NP_/CeO_2_, and the morphology remains flat for CeO_2_–Ar (Fig. S7). For Os_NP_, the carbon fiber without CeO_2_ was directly used as the support for the deposition of Os. In contrast to Os_SA_–CeO_2_, Os aggregated into nanoparticles in both Os_NP_/CeO_2_ (Fig. S8) and Os_NP_ (Fig. S9). This indicates that the porous structure of the CeO_2_ matrix is crucial for obtaining atomically dispersed Os atoms due to its much larger exposed surface area (Fig. S10). In addition, the Os loading amount is also necessary for the successful formation of Os single atoms (Fig. S11).

**Fig. 3:**
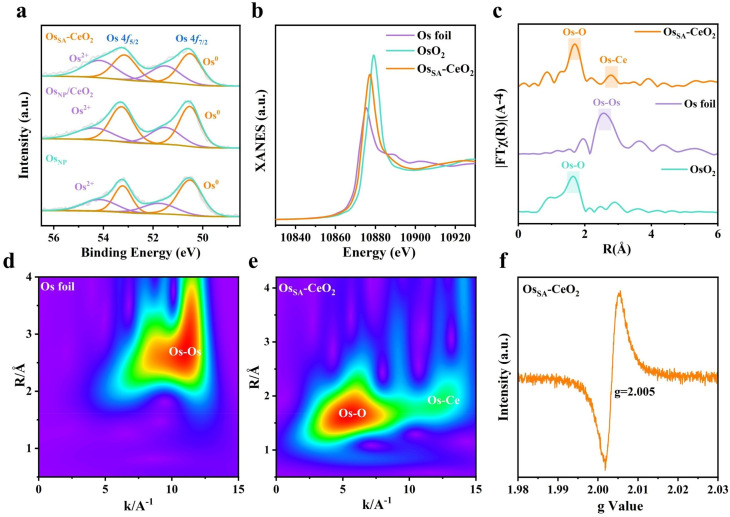
Structural characterization of Os_SA_–CeO_2_. (a) High-resolution XPS spectra of Os 4f. (b) Os L_3_-edge XANES spectra. (c) FT-EXAFS spectra of Os. WT of the *k*^4^-weighted EXAFS spectra of (d) Os_SA_–CeO_2_ and (e) Os foil. (f) EPR of Os_SA_–CeO_2_.

The electronic structure of Os in samples was investigated by X-ray photoelectron spectroscopy (XPS) and X-ray absorption near-edge structure (XANES) spectroscopy. In the Os 4f XPS spectra, the Os^2+^ fraction in Os_NP_/CeO_2_ is approximately 37 at%, which is a bit higher than the 36 at% for Os_NP_ (Fig. 3a). This is probably due to the electronic transfer between Os_NP_ and the CeO_2_ support.^31,32^ In contrast, Os_SA_–CeO_2_ exhibits a substantially increased Os^2+^ ratio of 46 at%. As revealed in Os L_3_ edge XANES spectra, Os in Os_SA_–CeO_2_ possesses an oxidation state between those of metallic Os and OsO_2_ (Fig. 3b). The much higher valence state of Os in Os_SA_–CeO_2_ further evidences the successful doping of Os into CeO_2_ that enables strong electronic interactions between them. Also, the relatively lower valence of Os compared to Ce^4+^ will induce the generation of oxygen vacancies to keep the electronic balance,^34^ consistent with the DFT results.

We studied the extended X-ray absorption fine structure (EXAFS) spectra to determine the coordination environments of Os in Os_SA_–CeO_2_. The Os L-edge R-space spectrum of Os_SA_–CeO_2_ (Fig. 3c) shows a dominant peak at ∼1.72 Å corresponding to the Os–O band, and no peak of Os–Os bond is observed. The small peak at ∼2.83 Å is probably ascribed to the Os–Ce bond,^24,35^ as illustrated by the Fourier transform (FT) EXAFS fitting (Fig. S12 and Table S2). These verify the atomic dispersion of Os in CeO_2_, consistent with the STEM results. Wavelet transform (WT)-EXAFS analysis (Fig. 3d and e) further confirms the appearance of Os–O and Os–Ce bands in Os_SA_–CeO_2_. The Os–O coordination number (3.2, Table S2) is significantly lower than the Ce–O coordination (7.7) in CeO_2_, indicating abundant oxygen vacancies, as confirmed by the electron paramagnetic resonance (EPR, Fig. 3f) and O 1s spectrum (Fig. S13) of Os_SA_–CeO_2_. The shorter Os–Ce bond (2.83 Å *vs.* Ce–Ce 3.30 Å) reflects lattice compression, consistent with the STEM results (Fig. 2e). Thus, we can conclude that the porous CeO_2_ support enables the successful fabrication of atomically dispersed Os SAs, accompanied by the generation of numerous oxygen vacancies within the CeO_2_ matrix.

### Electrochemical performances

**Fig. 4:**
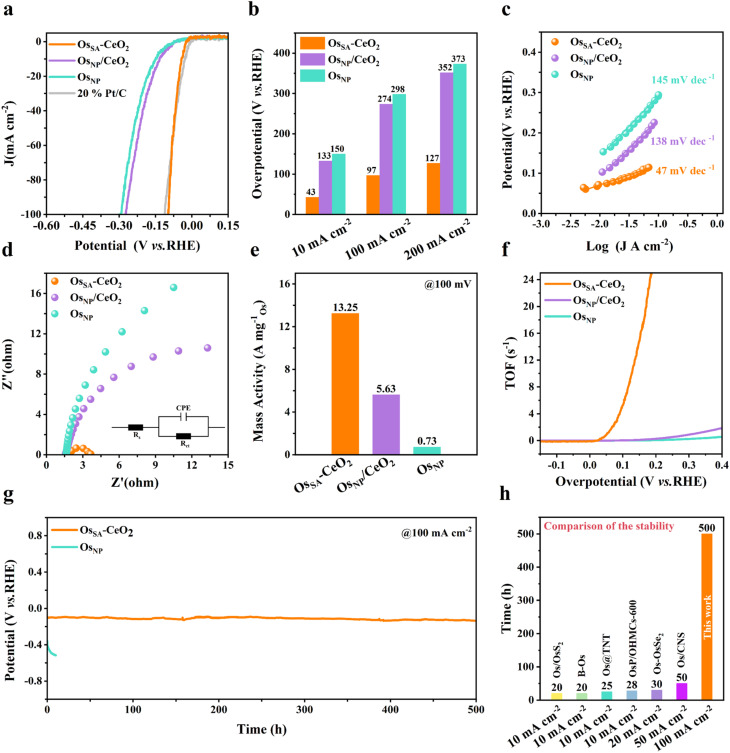
HER performance of Os_SA_–CeO_2_ in acidic media. (a) HER polarization curves of various catalysts in 0.5 M H_2_SO_4_. (b) Comparison of overpotentials at 10, 100 and 200 mA^−2^. (c) Tafel plots. (d) Nyquist plots; the inset shows the corresponding equivalent electrical circuit used for fitting. (e) Mass activity at an overpotential of 100 mV. (f) Relationship between TOF values and overpotentials. (g) Stability tests of Os_SA_–CeO_2_ and Os_NP_. (h) Comparison of the stability of Os_SA_–CeO_2_ with previously reported Os-based HER catalysts in acidic media.^14,15,18–21^

HER measurements were conducted in 0.5 M H_2_SO_4_ using a three-electrode setup. As shown in Fig. 4a, Os_SA_–CeO_2_ exhibits markedly superior HER activity compared to Os_NP_/CeO_2_ and Os_NP_, approaching the performance of commercial 20% Pt/C. The overpotentials of Os_SA_–CeO_2_ are only 43 mV and 97 mV at current densities of 10 and 100 mA cm^−2^, respectively (Fig. 4b). The Tafel slope (47 mV dec^−1^, Fig. 4c) is significantly lower than that of Os_NP_/CeO_2_ (139 mV dec^−1^) and Os_NP_ (145 mV dec^−1^), indicating faster kinetics and a favorable HER pathway.^36–38^ Electrochemical impedance spectroscopy (Fig. 4d) shows that Os_SA_–CeO_2_ possesses the lowest charge transfer resistance, as summarized in Table S3. These factors contribute to its exceptionally high mass activity (Fig. 4e) and TOF (Fig. 4f). The mass activity of Os_SA_–CeO_2_ is 13.25 A mg_Os_^−1^ at 100 mV overpotential (Fig. 4e), 2.4 times higher than that of Os_NP_/CeO_2_ and 18.2 times higher than that of Os_NP_ (Table S4). The effect of Os loading on HER performance was further investigated by varying the deposition cycles (Fig. S14). Os-50 CV (Os_SA_–CeO_2_) exhibits markedly higher activity than Os-10 CV, while showing slightly lower activity than Os-90 CV. However, excessive Os loading in Os-90 CV leads to the formation of large aggregated nanoparticles (Fig. S11c), which is detrimental to achieving atomically dispersed active sites. Therefore, Os-50 CV, which balances high catalytic activity with atomic dispersion, was selected as the representative sample for further investigation.

Post-HER XPS analysis (Fig. S15) reveals minimal changes in Os binding energy for Os_SA_–CeO_2_, whereas Os_NP_/CeO_2_ and Os_NP_ exhibit positive shifts, indicating Os oxidation. Due to the strong interactions between Os and CeO_2_ in Os_SA_–CeO_2_, the electron-buffering effect of CeO_2_ regulates the electronic structure of Os and prevents its oxidation dissolution.^33,39^ Controlled-current water electrolysis (Fig. 4g) was further done to test the long-term performance and stability. Os_SA_–CeO_2_ maintains stable operation for over 500 h at 100 mA cm^−2^, far exceeding Os_NP_ and previously reported Os-based HER catalysts (Fig. 4h).

### Insights into the origin of enhanced stability at large current densities

**Fig. 5:**
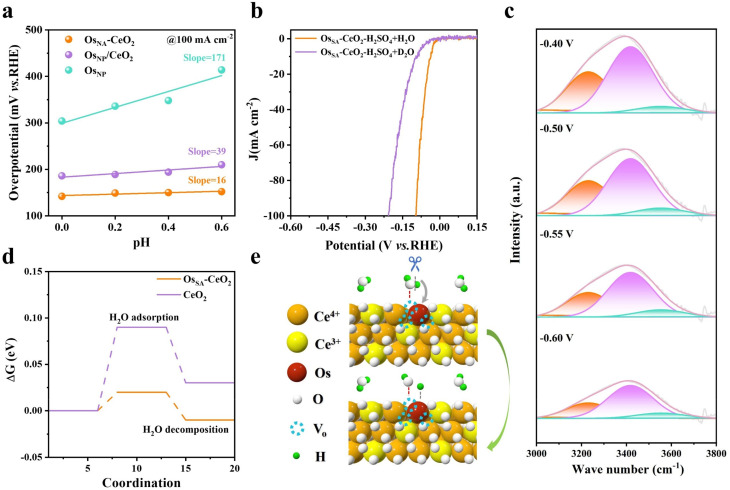
Mechanistic insights into the H_2_O splitting ability of Os_SA_–CeO_2_. (a) Overpotentials as a function of pH at100 mA cm^−2^ for Os_SA_–CeO_2_, Os_NP_/CeO_2_, and Os_NP_. (b) LSV curves of Os_SA_–CeO_2_ measured in 0.5 M H_2_SO_4_ + H_2_O and 0.5 M H_2_SO_4_ + D_2_O. (c) *In situ* FTIR spectroscopy of Os_SA_–CeO_2_ at different applied potentials. (d) Gibbs free energy diagrams for H_2_O adsorption and decomposition on Os_SA_–CeO_2_ and CeO_2_. (e) Schematic illustration of the HER reaction pathway, showing H_2_O as the supplied hydrogen source.

pH-dependent experiments (Fig. S16 and 5a) were performed to simulate the insufficient proton supply conditions that occur during high current operation. The overpotential at 100 mA cm^−2^ increases sharply with pH for Os_NP_ (slope = 171), indicating strong proton dependence, whereas Os_SA_–CeO_2_ shows a much smaller slope (16), suggesting additional water-splitting participation. Kinetic isotope effect (KIE) experiments (Fig. 5b) confirm this: HER performance in 0.5 M H_2_SO_4_ with D_2_O is significantly lower than in 0.5 M H_2_SO_4_ with H_2_O, due to slower D^+^ migration and higher D–D bond energy, implying that H_2_O dissociation contributes to H* generation on Os_SA_–CeO_2_. *Operando* infrared spectroscopy (Fig. 5c) further supports this conclusion. The broad band from 3000–3800 cm^−1^ corresponds to interfacial water species, including 4-hydrogen bonding (HB)-H_2_O (3231 cm^−1^), 2-HB-H_2_O (3418 cm^−1^), and free H_2_O (3553 cm^−1^). With increasing negative potential, the proportion of strongly bound 4-HB-H_2_O decreases, transforming into free H_2_O (Table S5), indicating active water participation during the HER.^40^

The facile participation of H_2_O for the HER on Os_SA_–CeO_2_ is probably due to the abundant oxygen vacancies. The oxygen vacancies can act as the strong adsorption sites for the oxygen-containing species like H_2_O, which is confirmed by the DFT calculations (Fig. 5d). The Gibbs free energy barrier (Δ*G*) for H_2_O adsorption decreases from 0.09 eV on CeO_2_ to 0.02 eV on Os_SA_–CeO_2_, greatly facilitating H_2_O dissociation. As summarized in Fig. 5e, Os doping induces oxygen vacancies that promote H_2_O adsorption and splitting. The generated H* species migrate to adjacent Os sites, serving as a hydrogen source to support the rapid proton consumption at large current densities, thereby enabling stable HER operation. Also, the generated OH* species are adsorbed by the oxygen vacancies, thus preventing the Os sites from oxidative dissolution.

## Conclusions

In summary, we have successfully synthesized an Os_SA_–CeO_2_ catalyst composed of atomically dispersed Os single atoms strongly anchored on a porous CeO_2_ support. The porous CeO_2_ architecture provides abundant anchoring sites that stabilize Os atoms and strengthen the Os–CeO_2_ interfacial coupling. This interaction optimizes the electronic structure of Os, moderates H* binding strength, and induces abundant oxygen vacancies that activate CeO_2_ toward enhanced H_2_O dissociation. Benefiting from these synergistic effects, the Os_SA_–CeO_2_ catalyst exhibits outstanding HER activity in acidic electrolytes, surpassing commercial Pt/C at high current densities. It delivers a remarkable mass activity of 13.25 A mg^−1^ at 100 mV overpotential and maintains stable operation for over 500 h at 100 mA cm^−2^ with negligible degradation. This work provides a new design strategy to enhance the stability of noble-metal catalysts without sacrificing activity by engineering strong metal–support interfacial interactions.

## Author contributions

J. Y. conceptualized the project. J. Y. and L. Z. supervised the work. Q. L. performed catalyst synthesis and general characterization. Q. L., M. L., W. L., and Y. H. contributed to experimental design and data analysis. Q. L. and J. Y. wrote the original manuscript draft. All authors contributed to discussions on the data and to the development of the manuscript.

## Conflicts of interest

There are no conflicts to declare.

## Supplementary Material

SC-017-D5SC09741J-s001

## Data Availability

The data supporting this article have been included as part of the supplementary information (SI). Supplementary information: experimental methods, additional DFT calculations, structure models for DFT calculations; SEM, TEM, schematic illustration of the synthesis route, EXAFS, XPS and ICP-OES; additional electrochemical data and summarized table for situ FTIR spectroscopy. See DOI: https://doi.org/10.1039/d5sc09741j.
