# Supplementary material for: Atomic-level interface engineering enables efficient and durable acidic hydrogen evolution of osmium at large current densities
Source: Chem Sci. 2026 Feb 10;17(14):6956–63. doi: 10.1039/d5sc09741j (PMC12908458; doi:10.1039/d5sc09741j)
Supplement: SC-017-D5SC09741J-s001 [file SC-017-D5SC09741J-s001.pdf]

Supplementary Information (SI) for Chemical Science.
This journal is © The Royal Society of Chemistry 2026

Atomic-level interface engineering enables efficient and durable acidic hydrogen evolution of osmium under large current densities

Qianyi Lin, Jun Yu,* Mansheng Liao, Weidong Liang, Yayun Hong, Huiqi Li, Zhongxin Song, and Lei Zhang*

College of Chemistry and Environmental Engineering, Shenzhen University, Shenzhen 518071, China. E-mail: yujun@szu.edu.cn; lei.zhang@szu.edu.cn

Experiment Section:

Materials: All chemicals, including Osmium(IV) chloride (K_2OsCl_6, 38.7%, Macklin), cerium(III) hexahydrate ($Ce(NO_3)_3 \cdot 6H_2O$, 99%, Macklin), HCl (36-38%, Shenzhen Boleen Technology Co., Ltd.), absolute alcohol (98%, Aladdin), Millipore water (0.07 μS cm^{-1}), and Carbon paper (CP) (TGPH060, Toray), were used without further purification.

Synthesis of Os_{SA}-CeO_2, Os_{NP}/CeO_2, and Os_{NP} samples: CeO_2 support: carbon paper (2 × 2 cm) was ultrasonically cleaned in deionized water (10 min), HCl (90 min), and ethanol (10 min). The CeO_2 plating solution was prepared by dissolving $Ce(NO_3)_3 \cdot 6H_2O$ (0.88 g, 2 mM) and NaCl (0.59 g, 10 mM) in deionized water (1 L). A 50 mL aliquot was used in a PTFE cell with carbon paper (working electrode), graphite rod (counter), and Ag/AgCl (reference). Electrodeposition was performed at 1 mA for 900 s while the plating cell was placed in a 70 °C hot-water bath. The obtained films were calcined at 500 °C (5 °C·min^{-1}) for 2 h. Os single-atom incorporation: the obtained CeO_2/carbon paper was cut into 1 × 2 cm pieces, and a 1 × 1 cm working area was defined using sealing film. Prior to deposition, the electrode was activated at −100 mA·cm^{-2} for 20 min. For Os incorporation, 962 μL of 1 mg·mL^{-1} K_2OsCl_6 was added to 40 mL of 0.5 M K_2SO_4. Electrodeposition was conducted using CeO_2/carbon paper as the working electrode, a carbon rod counter electrode, and an RHE reference. Specifically, cyclic voltammetry (CV) was applied for 50 cycles within a potential window of 0 to −0.4 V vs. RHE, corresponding to a total deposition time of approximately 13 minutes. For Os_{NP}/CeO_2 and Os_{NP} samples, the Os deposition process is identical to that used for Os_{SA}-CeO_2. The substrates employed are CeO_2 annealed in Ar (CeO_2-Ar) for Os_{NP}/CeO_2 and carbon paper for Os_{NP}, respectively.

Structural characterization: The morphologies of the prepared samples were examined using field-emission scanning electron microscopy (FESEM, JSM-7800F) and transmission electron microscopy (TEM, JEM-F200). High-resolution structural characterization, single-atom identification, and elemental mapping were performed using a spherical aberration–corrected TEM (Thermo Fisher Spectra 200). The crystal

phases were analyzed by X-ray diffraction (XRD, Empyrean). The surface chemical states were determined by X-ray photoelectron spectroscopy (XPS, Thermo Scientific Nexsa). In situ Fourier-transform infrared (FTIR) spectroscopy was carried out using a CHI 760e electrochemical workstation (Shanghai, China) equipped with a three-electrode system. The sample served as the working electrode, Hg/HgO as the reference electrode, and a carbon rod as the counter electrode. This setup was used to monitor variations in the hydrogen-bond network strength during the cathodic hydrogen evolution reaction (HER) in water electrolysis. Elemental contents were determined by inductively coupled plasma optical emission spectrometry (ICP-OES, Avio 200). X-ray absorption fine structure (XAFS) measurements were conducted at the Os L_3-edge on beamline BL14W1 at the Shanghai Synchrotron Radiation Facility (SSRF, Shanghai, China), using Si(111) crystal monochromators. Before analysis, samples were pressed into 1 cm diameter pellets and sealed with Kapton tape. The spectra were collected in fluorescence mode at room temperature using a four-channel silicon drift detector (SDD, Bruker 5040). Negligible variations in line shape or peak position were observed between repeated scans for a given sample. The spectra were processed and analyzed using the Athena and Artemis software packages.

Electrochemical measurements: Electrochemical measurements were performed at room temperature in 0.5 M H_2SO_4 electrolyte using a standard three-electrode configuration, with Os_{SA}-CeO_2 as the working electrode, a carbon rod as the counter electrode, and a reversible hydrogen electrode (RHE) as the reference electrode. Linear sweep voltammetry was carried out at a scan rate of 5 mV/s. Electrochemical impedance spectroscopy (EIS) was measured over a frequency range of 10^{-1} to 10^5 Hz. The double-layer capacitances (Cdl) of the samples were evaluated by recording cyclic voltammetry (CV) curves in the non-Faradaic potential region (0.51-0.63 V vs. RHE) at scan rates of 0.02, 0.04, 0.06, 0.08, and 0.10 mV/s. Tafel plots were obtained using a scan rate of 1 mV/s. All tests were conducted under stagnant electrolyte conditions.

TOF: Turnover frequency (TOF) was calculated as the following equation:

$$TOF = \frac{j \times A}{2 \times F \times n}$$

j is the measured current density at given overpotential. A is the area of the electrode. F is the Faraday constant (96485 C/mol). n is the number of all metal atoms that are coated onto the electrode, and the specific content is obtained by ICP.

DFT calculations: First-principles DFT calculations were performed using the projector-augmented wave method and the Perdew–Burke–Ernzerhof exchange-correlation functional as implemented in the Vienna Ab Initio Simulation Package (VASP).[1-4] The $2\sqrt{3} \times 3$ supercell with twelve atomic layers was constructed to simulate the (111) and Os-doped (111) CeO_2 surfaces, the atoms of the middle four layers were fixed, and the rest were allowed to relax. A vacuum distance of 18Å was found to be adequate. The cut-off energy for plane wave was set to 520 eV after a series of test, and the convergence criterion for the residual force and energy was set to 0.02 eV $Å^{-1}$ and 10^{-4} eV, respectively. The empirical correction in Grimme's method (DFT+D3) was used to describe the van der Waals (vdW) interactions.[5] To understand the role of f electrons in determining the rare-earth oxide band gaps, the models were explored using DFT+U with an effective U-J term of 6.0 and 5.0 eV and for Os and Ce elements.[6] The Brillouin region was sampled by the Monkhorst-Pack method with a 3×3×1 k-point mesh.[7] Here, differences in Gibbs free energies (ΔG) for intermediates on catalysts surface involved in the acid hydrogen evolution process were calculated by using the computational hydrogen electrode model,

$$\Delta G = \Delta E + \Delta E_{ZPE} - T\Delta S + \Delta G_U$$

where the reaction energy (ΔE) can be directly obtained by analyzing the DFT total energies. The zero-point energy difference (ΔE_{ZPE}) between the products and the reactants can be computed from the vibrational frequencies. $\Delta S = S(H^*) - 1/2 \cdot S(H_2)$ is the change in entropy between the products (H_2) and the reactants (H^*) at room temperature (T=298.15 K), and the latter is approximately zero. $\Delta G_U = -eU$, whereby U is the electrode potential. All the structures discussed in this study were rendered by VESTA.[8]

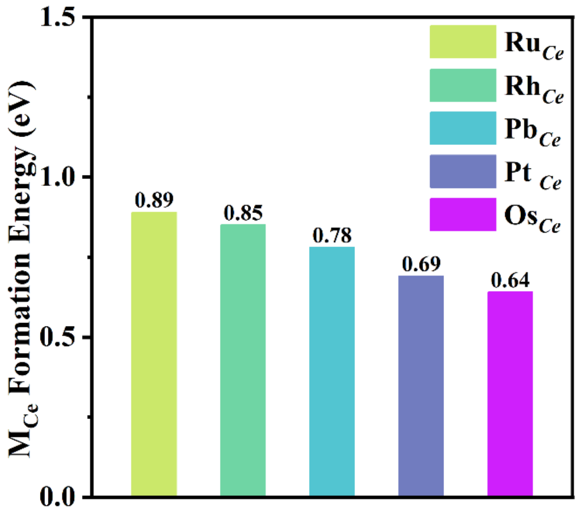

Fig. S1 Calculated formation energies of M_{Ce} (M stands for the substituted atom for Ce).

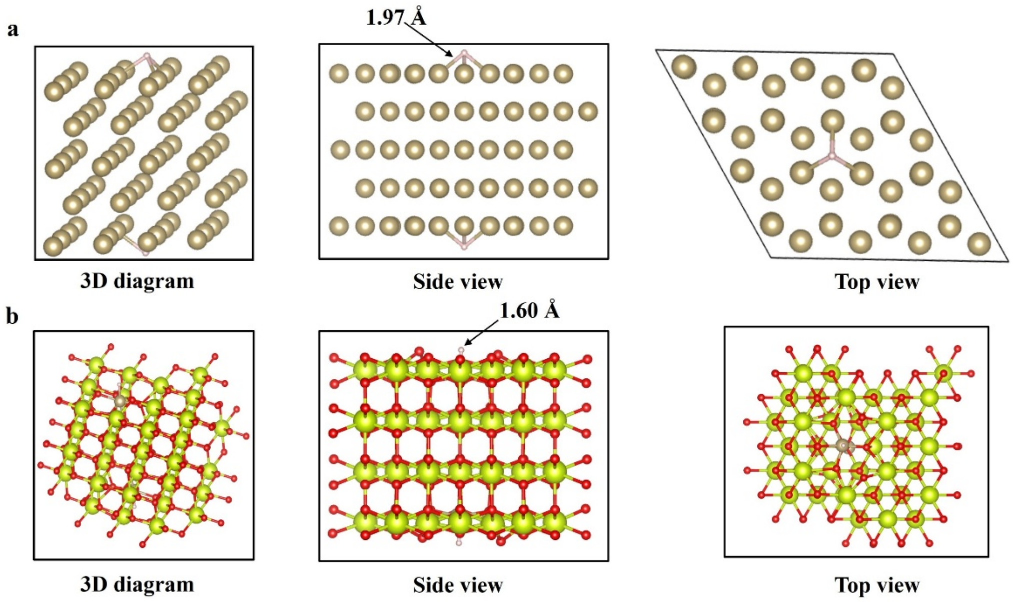

Fig. S2 The key HER intermediate of H* adsorbed on Os site of a) Os_{NP} and b) O_{SA}-CeO_2. The silver, yellow and red spheres represent Os, Ce and H atoms, respectively.

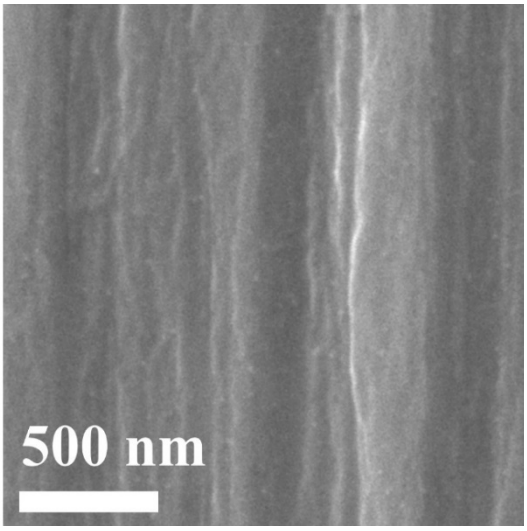

Fig. S3 SEM image of CeO_2.

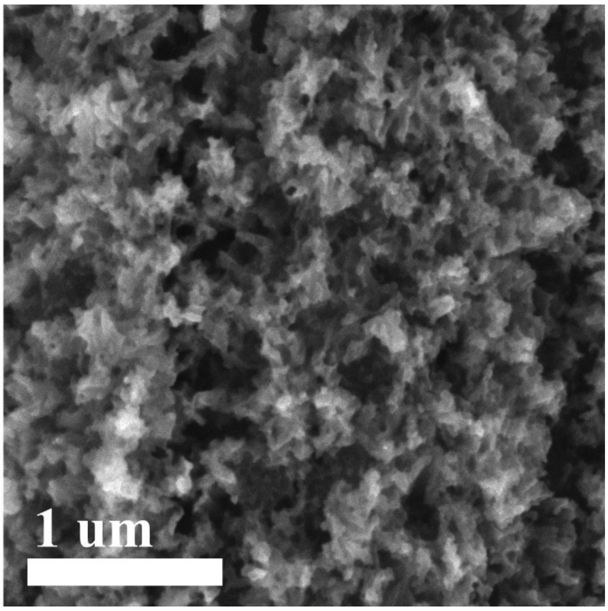

Fig. S4 SEM image of CeO_2-O_2.

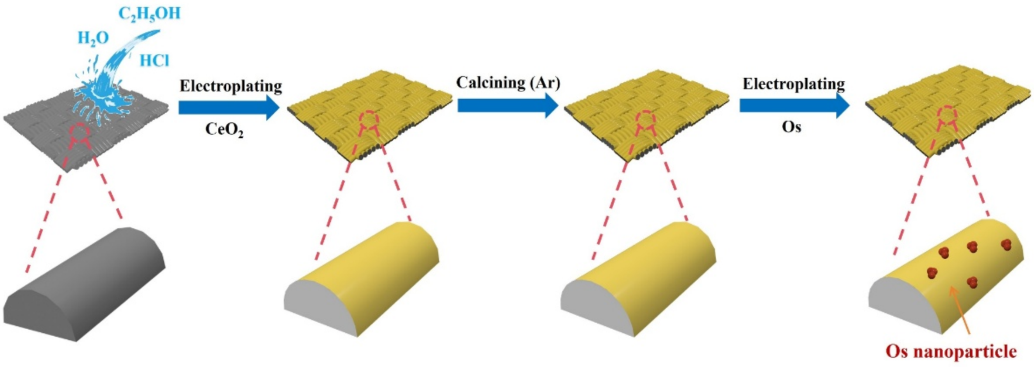

Fig. S5 Schematic illustration of the synthesis route for Os_{NP}/CeO_2.

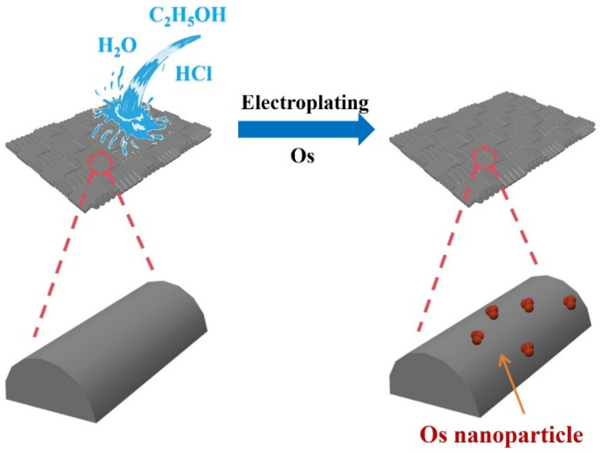

Fig. S6 Schematic illustration of the synthesis route for Os_{NP}.

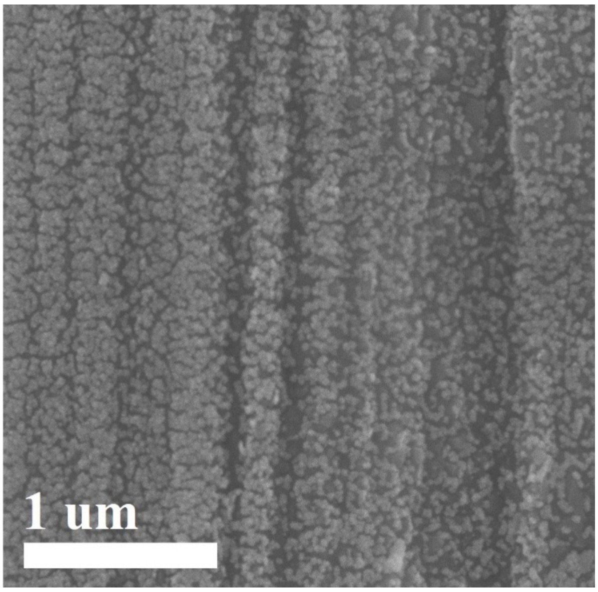

Fig. S7 SEM image of CeO_2-Ar.

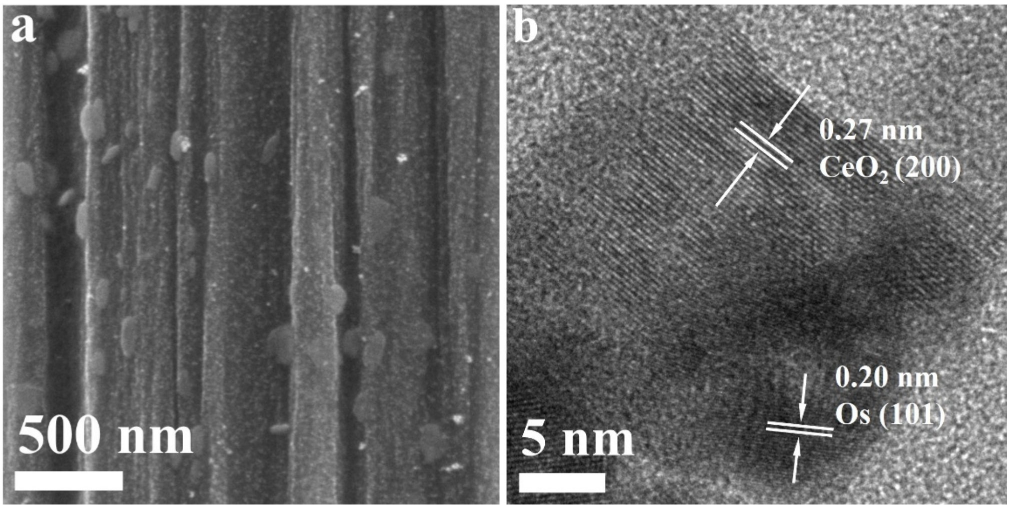

Fig. S8 (a) SEM image and (b) TEM image of Os_{NP}/CeO_2.

Abundant nanoparticles are observed in the SEM image of Os_{NP}/CeO_2 (Fig. S8a), and TEM analysis (Fig. S8b) further confirms that these particles are metallic Os.

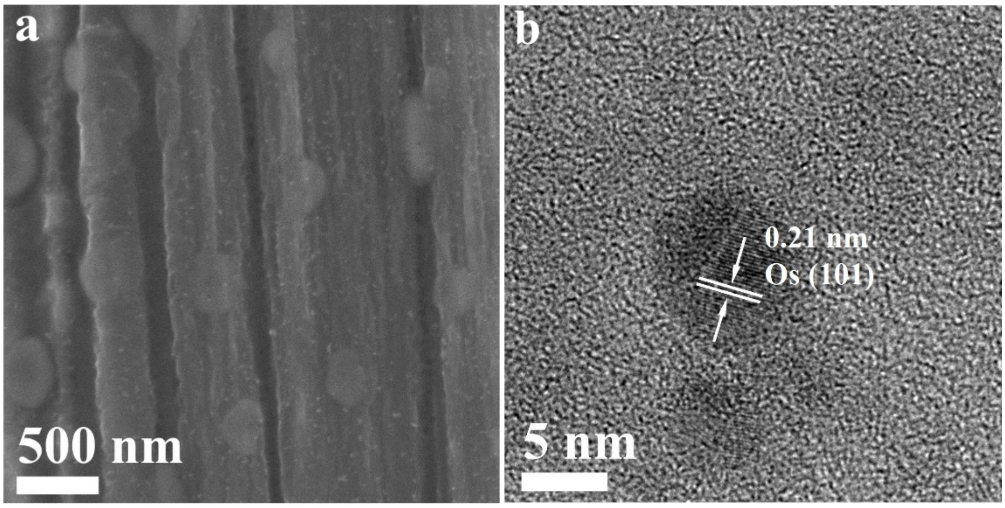

Fig. S9 (a) SEM image and (b) TEM image of Os_{NP}.

Abundant nanoparticles are observed in the SEM image of Os_{NP} (Fig. S9a), and TEM analysis (Fig. S9b) further confirms that these particles are metallic Os.

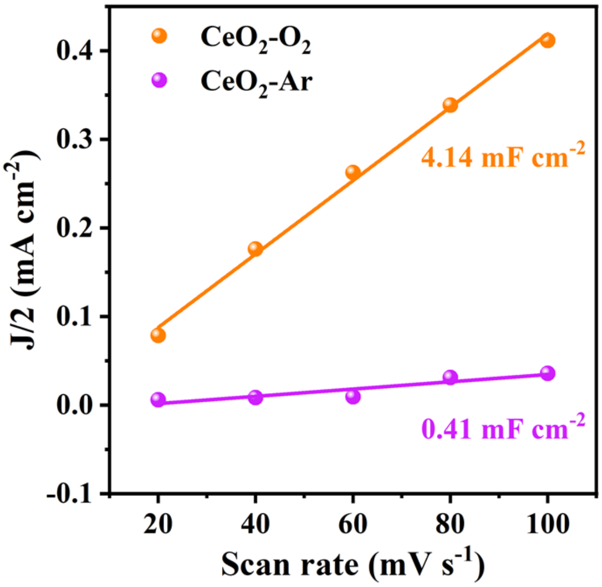

Fig. S10 Electrochemical active surface area (ECSA) of CeO_2-O_2 and CeO_2-Ar.

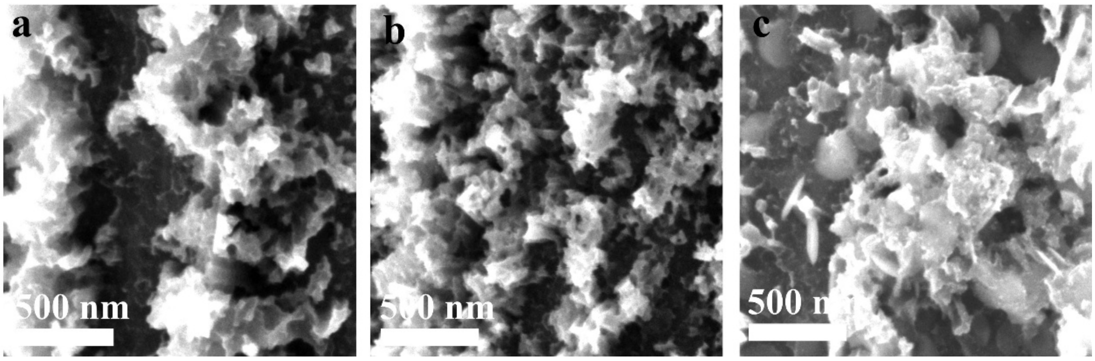

Fig. S11 SEM images of (a) Os-10 CV, (b) Os-50 CV (Os_{SA}-CeO_2), and (c) Os-90 CV.

To tune the amount of Os deposited on porous CeO_2, different numbers of CV scan cycles were applied during the electrodeposition process. As shown in Fig. S11, the morphology of the sample with 10 CV scans (Os-10 CV) is very similar to that of the Os_{SA}-CeO_2 sample prepared with 50 CV scans. However, when the number of CV scans increases to 90 (Os-90 CV), a large number of nanoparticles are formed.

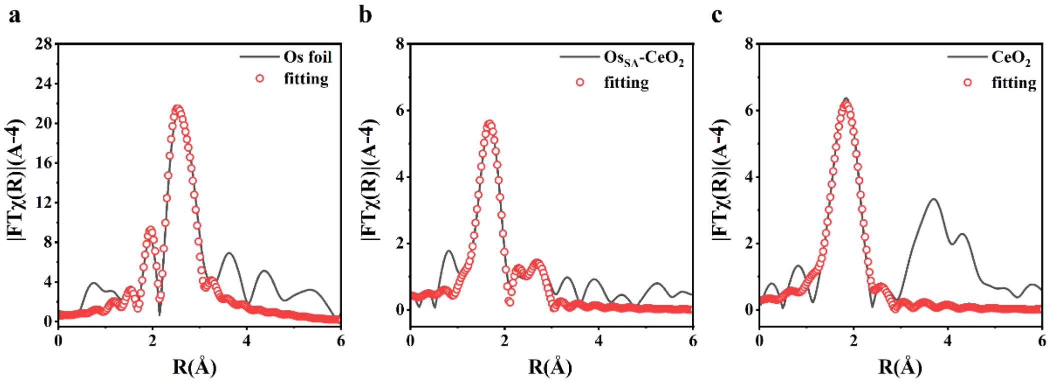

Fig. S12 Fourier transform (FT) EXAFS fitting of Os in a) Os foil and b) Os_{SA}-CeO_2. c) FT EXAFS fitting of Ce in CeO_2.

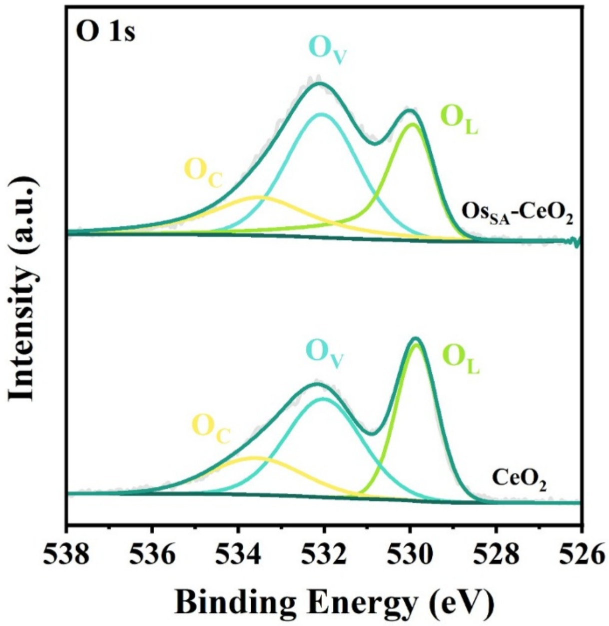

Fig. S13 High-resolution XPS spectra of O 1s.

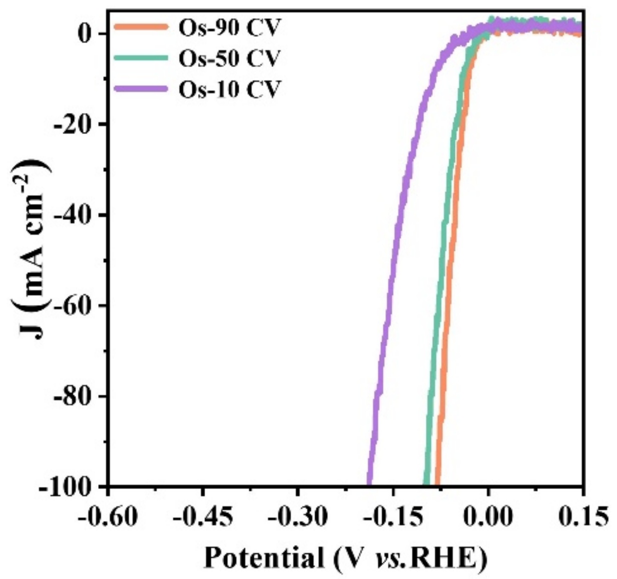

Fig. S14 HER polarization curves of Os-10 CV, Os-50 CV (Os_{SA}-CeO_2) and Os-90 CV.

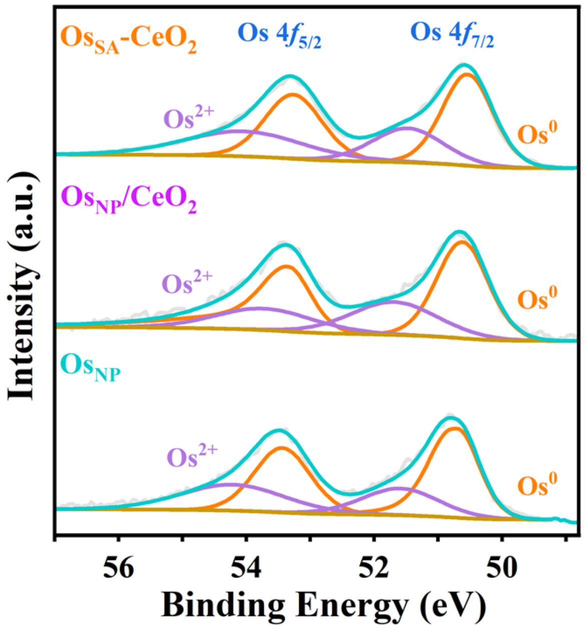

Fig. S15 High-resolution XPS spectra of Os_{SA}-CeO_2, Os_{NP}/CeO_2 and Os_{NP} after HER reaction.

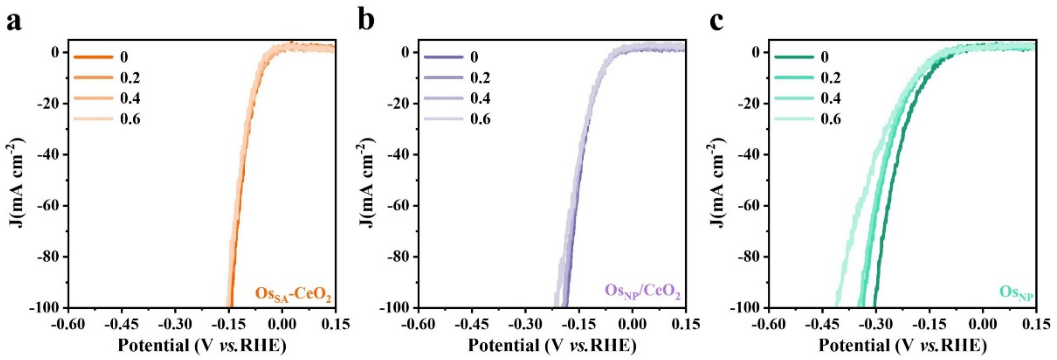

Fig. S16 LSV curves of a) Os_{SA}-CeO_2, b) Os_{NP}/CeO_2 and c) Os_{NP} under different pH conditions.

Table S1. Contents of Os and Ce in Os_{SA}-CeO_2 determined from HAADF-STEM.

Element	Content (%)
Os	0.09
Ce	0.32

Table S2. EXAFS fitting parameters at the L_3-edge of Ce and Os.

Sample	Shell	CN^a	R(Å)^b	σ^2(Å^2)^c	ΔE_0(eV)^d	R factor
CeO_2	Ce-O	7.7±1.0	2.27±0.01	0.01	6.2	0.0198
Os foil	Os-Os	12	2.70±0.01	0.0044	9.2	0.0114
OsO_2	Os-O	4	1.66±0.01	0.0078	14	0.0174
Os_{SA}-CeO_2	Os-O	3.2±0.2	1.72±0.01	0.0065	14.3	0.0194
	Os-Ce	1.0±0.3	2.83±0.01	0.008	6.8	

Table S3. The EIS fitting results of catalysts at 0 V vs. RHE.

Catalyst	R_{CT} Ω	Error %
Os_{SA}-CeO_2	1.6	0.4
Os_{NP}/CeO_2	23.5	1.4
Os_{NP}	51.4	1.5

Table S4. Contents of Os in Os_{SA}-CeO_2, Os_{NP}/CeO_2 and Os_{NP} determined from ICP.

Catalyst	Os_{SA}-CeO_2	Os_{NP}/CeO_2	Os_{NP}
Os (μg/cm^2)	7.925	0.775	2.325

Table S5. Proportions of different interfacial water species at various potentials obtained from in situ FTIR spectroscopy.

Potential (V)	4-HB-H_2O (%)	2-HB-H_2O (%)	free-H_2O (%)
-0.4	34.82	59.14	6.04
-0.5	33.84	58.17	7.99
-0.55	30.84	59.29	9.87
-0.60	27.59	62.23	10.18

References

1. Kresse, G. and Furthmüller, J., *Comput. Mater. Sci.*, 1996, **6**, 15–50.
2. Kresse, G. and Furthmüller, J., *Phys. Rev. B*, 1996, **54**, 11169–11186.
3. Perdew, J. P., Burke, K. and Ernzerhof, M., *Phys. Rev. Lett.*, 1996, **77**, 3865–3868.
4. Kresse, G. and Joubert, D., *Phys. Rev. B*, 1999, **59**, 1758–1775.
5. Grimme, S., Antony, J., Ehrlich, S. and Krieg, H., *J. Chem. Phys.*, 2010, **132**, 154104.
6. Cococcioni, M. and de Gironcoli, S., *Phys. Rev. B*, 2005, **71**, 035105.
7. Monkhorst, H. J. and Pack, J. D., *Phys. Rev. B*, 1976, **13**, 5188–5192.
8. Momma, K. and Izumi, F., *J. Appl. Crystallogr.*, 2011, **44**, 1272–1276.
